# Leveraging videos and community health workers to address social determinants of health in immigrants (LINK-IT): Protocol for a randomized controlled trial

**DOI:** 10.1371/journal.pone.0341217

**Published:** 2026-02-02

**Authors:** Lu Hu, Jing Liu, Ximin Yang, Crystal Teng, Huilin Li, Yanan Zhao, Natalie Levy, Kelly Zhu, Suzanne Vang, Simona C. Kwon, Naumi Feldman, Jennifer Lau, Yanping Jiang, Chau Trinh-Shevrin, Nadia Islam

**Affiliations:** 1 Department of Population Health, NYU Grossman School of Medicine, NYU Langone Health, New York, New York, United States of America; 2 Department of Medicine, NYU Grossman School of Medicine, NYU Langone Health, New York, New York, United States of America; 3 Charles B. Wang Community Health Center, Inc., New York, New York, United States of America; 4 Institute for Health, Health Care Policy and Aging Research, Rutgers, The State University of New Jersey, New Brunswick, New Jersey, United States of America; 5 Department of Family Medicine and Community Health, Rutgers, The State University of New Jersey, New Brunswick, New Jersey, United States of America; PLOS: Public Library of Science, UNITED KINGDOM OF GREAT BRITAIN AND NORTHERN IRELAND

## Abstract

**Background:**

Chinese immigrants face numerous social determinants of health (SDOH) challenges that limit access to evidence-based diabetes self-management education and support programs (DSMES). To address these challenges, our team developed the LINK-IT intervention. This manuscript presents the study protocol for the LINK-IT trial.

**Methods:**

The LINK-IT trial is a 12-month, 3-arm randomized controlled trial aiming to enroll 405 Chinese immigrants with T2D (HbA1c≥7%) from multiple community and clinical settings in New York City. A total of 405 participants will be randomly allocated to one of three groups (n = 135 per group): (1) video-based DSMES plus community health worker (CHW) support (VIDEO+CHW), (2) video-based DSMES only (VIDEO), or (3) wait-list control (CONTROL). The VIDEO+CHW group will receive 24 culturally and linguistically tailored DSMES videos (one per week for 24 weeks) delivered via text message links, along with biweekly (every other week) phone calls from trained CHWs to review video content, support goal setting, and address SDOH barriers. The VIDEO group will receive the same video intervention without CHW support. The CONTROL group will receive usual care and will be offered access to the videos upon study completion. The primary outcome is the change in HbA1c at 6 months. Secondary outcomes include changes in HbA1c at 12 months, self-efficacy for diabetes, dietary intake, physical activity, medication adherence and emotional support at 6 and 12 months. Data will be analyzed using an intention-to-treat approach with linear mixed-effects models.

**Ethics and Dissemination:**

This study protocol has been approved by the Institutional Review Board of the NYU Grossman School of Medicine (S23-01274). All study procedures will adhere to the ethical principles outlined in the Declaration of Helsinki. Written or verbal informed consent will be obtained from all participants. Study results will be disseminated through peer-reviewed publications, presentations at scientific conferences, and community events.

**Trial registration:**

The LINK-IT trial was registered on March 20, 2024, on ClinicalTrials.gov under the identifier NCT06319716; https://clinicaltrials.gov/study/NCT06319716.

## Introduction

Asian Americans are the fastest-growing racial minority group in the United States, with Chinese immigrants representing the largest Asian subgroup, accounting for nearly 22% of the Asian American population [1,2]. Chinese immigrants face a disproportionate but often overlooked burden of type 2 diabetes (T2D) [3–5]. For example, New York City (NYC) is home to approximately 630,000 Chinese Americans, and alarmingly, nearly one in two Chinese immigrants in the city has T2D or prediabetes [3]. A significant proportion of Chinese immigrants with T2D live in low-income households, have limited English proficiency (LEP) (nearly 60%), and experience suboptimal glycemic control and self-management behaviors [6–8].

Diabetes Self-Management Education and Support (DSMES) programs are cornerstone interventions for improving glycemic control and self-management behaviors [9]. However, access to DSMES remains severely limited for Chinese immigrants due to multiple barriers, including linguistic and cultural discordance with healthcare providers, the intensive time commitment required for in-person sessions, and social determinants of health (SDOH) barriers such as lack of health insurance and financial constraints [10–14]. Mobile health (mHealth) interventions leveraging widely used technologies, including text messaging, offer a promising avenue to increase access [15–17]. Our pilot work demonstrated high feasibility and acceptability of a text-message-delivered, video-based DSMES intervention in Chinese immigrants with T2D [18–20]. However, technology-only interventions are often insufficient to address complex social needs that create barriers to effective diabetes management [15–17]. The National Institute on Minority Health and Health Disparities (NIMHD) Research Framework emphasizes the importance of multicomponent interventions addressing SDOH in medically underserved populations, such as immigrants [21].

The community health worker (CHW) model has demonstrated effectiveness in addressing social needs and providing culturally congruent support for chronic disease management in medically underserved communities [22–24]. Researchers have increasingly advocated for integrating CHWs into intervention strategies to enhance their impact by mitigating SDOH-related barriers to care among specific populations, including immigrants with LEP [22–26]. Building on this body of evidence and informed by our pilot work [18–20], we incorporated a CHW component into the culturally and linguistically tailored video-based DSMES intervention and developed the LINK-IT (Leveraging vIdeos and commuNity health worKers to address socIal determinants of health in immigranTs) project, aiming to provide comprehensive support to assist Chinese immigrants in managing their diabetes and improving health outcomes. This manuscript describes the study protocol for the LINK-IT project.

## Methods

### Study aim

This study aims to test the efficacy of two mHealth-based intervention strategies in improving diabetes outcomes among Chinese immigrants with poorly controlled T2D using a 3-arm randomized controlled trial (RCT). We hypothesize that the video-based DSMES intervention paired with CHW support will lead to the greatest reduction in HbA1c and improvements in psychosocial and behavioral outcomes, followed by the video-based DSMES intervention alone, and then the usual care group.

### Study design

**Fig 1:**
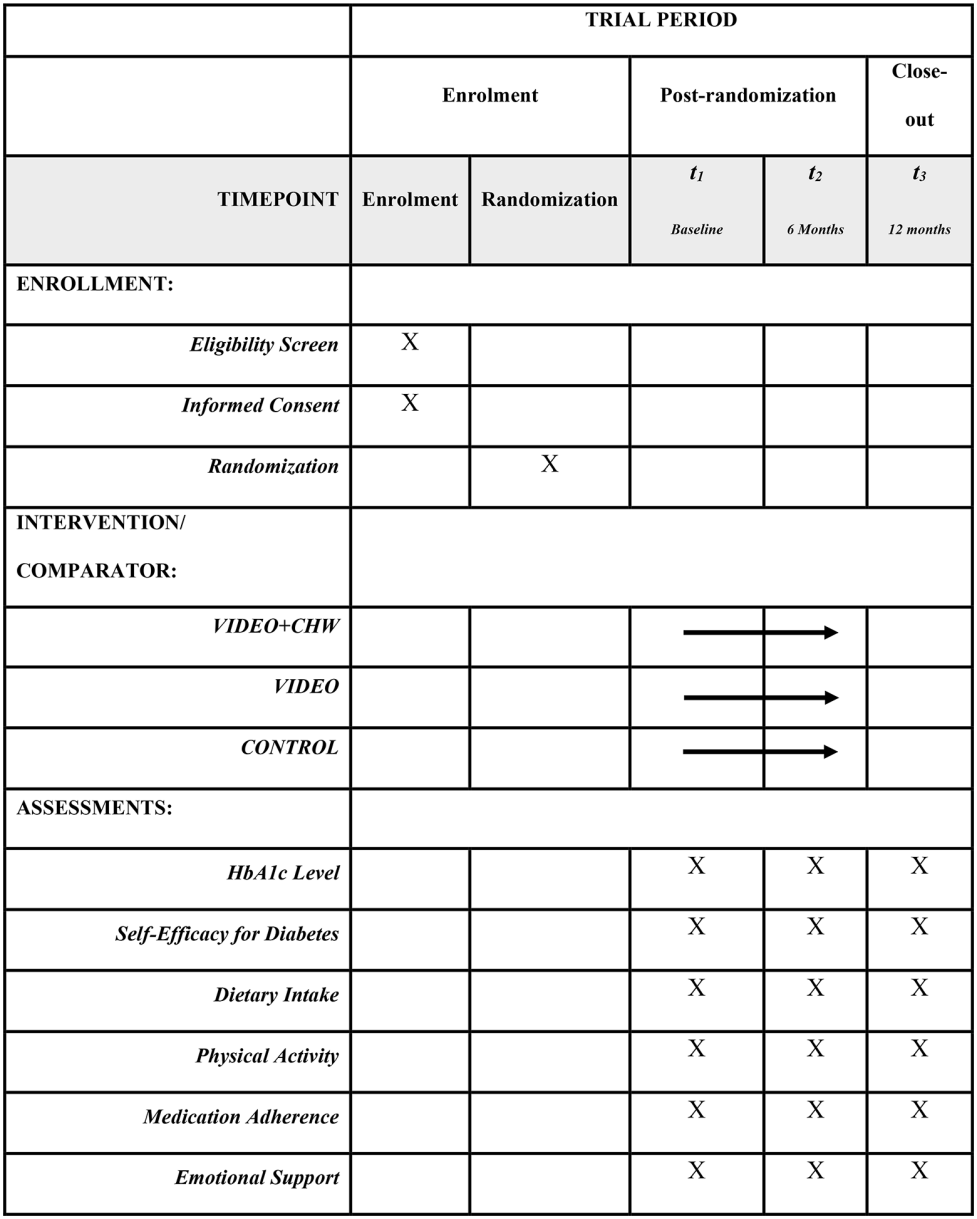
Participant timeline: Schedule of enrolment, interventions, and assessments.

The LINK-IT Project is a 5-year research initiative funded by the NIH from September 2023 to June 2028. Its efficacy will be evaluated through a 12-month 3-arm RCT. A total of 405 eligible participants will be randomized in equal allocation to one of three groups (n = 135 per group): (1) video-based DSMES plus CHW support (VIDEO+CHW), (2) video-based DSMES only (VIDEO), or (3) wait-list control (CONTROL). Outcomes will be assessed at baseline, 6 months, and 12 months. This trial protocol is reported in accordance with the SPIRIT 2025 Statement: Updated Guideline for Protocols of Randomized Trials guideline [27]. Fig 1 presents the SPIRIT schedule of enrollment, interventions, and assessments of the current trial.

### Study setting

We will recruit 405 eligible participants in NYC. Potential participants will be identified through electronic health records (EHR) and provider referrals across several clinic-based settings in NYC, including:

- Faculty group practices affiliated with a large academic medical system (NYU Langone Health);
- Federally qualified health centers (FQHCs), including the Charles B. Wang Community Health Center (CBWCHC), which serves Chinese immigrants in Manhattan and Queens Chinatown;
- Independent, community-based primary care practices serving Chinese populations.

Study flyers will also be distributed at these sites to facilitate recruitment. Our research team has maintained longstanding collaborations with these organizations for participant recruitment since 2016 through prior studies [18–20].

### Sample size calculation

The sample size was estimated based on the group difference in the change in HbA1c at 6 months, the primary outcome of this trial. Based on our preliminary data, we expect to observe a 0.5% difference in HbA1c change between the VIDEO+CHW and CONTROL groups [19]. Based on data from a prior CHW intervention in Hispanic immigrants with T2D, we expect to see a group difference of 0.4% in HbA1c change between the VIDEO+CHW and VIDEO groups [24]. Using the observed SD = 0.8% of HbA1c change in our preliminary study [19], a two-sample, two-sided t-test indicates that 86 participants per group will be required to detect a minimum group difference of 0.4% in HbA1c change with a power of 80% and a type I error of 0.0167 (to account for 3 group comparisons). We will extract HbA1c data from the EHR, which often has a high rate of missing data due to various reasons (e.g., changing to a different health care system). Our pilot study achieved a 70% completion rate of HbA1c follow-up data [19]. With a conservative completion rate of 60–65%, we will recruit 405 (135 per group) participants to yield a final sample of at least 258 (86 per group) to provide sufficient power to detect significant differences between the three group comparisons (VIDEO+CHW vs. VIDEO; VIDEO vs. CONTROL; VIDEO+CHW vs. CONTROL).

### Participant eligibility criteria

To be eligible for the study, participants must: (1) self-identify as ethnically Chinese, (2) be aged 18 years or older, (3) have a documented diagnosis of T2D in their medical record, (4) have an HbA1c≥7% in the past 12 months, (5) possess a smartphone or be willing to use a study-provided device, and (6) be willing to participate in video-based interventions.

Participants will be excluded if they are unable to provide informed consent, unable to participate meaningfully in the intervention (e.g., significant uncorrected visual or hearing impairments), are pregnant, planning a pregnancy, or are currently breastfeeding.

### Recruitment and informed consent

After identifying potential participants via EHR data search, the research team will first send a letter containing the study flyer to the participants’ addresses. Interested individuals can then contact the research team directly, after which a brief phone screening will be conducted to assess eligibility based on the study’s inclusion and exclusion criteria. During this call, research staff, who are bilingual in English and Chinese, will explain the study’s purpose, procedures, potential risks and benefits, and participants’ rights, ensuring that individuals have a clear understanding of the study before enrollment.

Those who meet all eligibility criteria and wish to participate will be scheduled for an enrollment visit at a convenient community location of their choice, where written informed consent will be obtained prior to any study procedures. To enhance accessibility and flexibility (e.g., for participants unable to attend in person due to long working hours), a verbal informed consent option will also be offered when appropriate.

### Randomization and blinding

Participants will be randomly assigned to one of the three study arms using a computer-generated permuted block randomization procedure (block size = 6), developed by the study biostatistics team. To minimize bias, data analysts and study investigators will remain blinded to participants’ group assignments. However, due to the nature of the interventions, neither participants nor CHWs delivering the intervention can be blinded.

### LINK-IT interventions

#### Overview.

The LINK-IT is a multicomponent intervention consisting of (1) 24 culturally and linguistically tailored DSMES videos delivered weekly for 24 weeks, and (2) CHW support calls delivered biweekly for 24 weeks.

The DSMES videos have been developed based on Social Cognitive Theory (SCT) [28], which posits that self-efficacy is a key determinant of behavioral change and is influenced by four major sources of information: mastery experiences, social modelling, verbal persuasion, and physiological states [28]. To promote mastery experiences, the intervention videos encourage participants to set incremental, achievable goals and to self-evaluate their progress. Participants are also counseled on using self-reward for goal achievement. The videos provide training in problem-solving strategies for common barriers to diabetes self-management. Social modelling is incorporated through videos featuring Chinese patients with similar immigration backgrounds who have successfully managed T2D. Verbal persuasion, which motivates behavior change, is delivered by CHWs, who reinforce the DSMES video content through physical diabetes counseling and by encouraging participants to adopt and maintain healthy behaviors, and participants are guided to recognize the physiological benefits of dietary modification and increased physical activity (e.g., improved sleep and better glucose control). In addition to content grounded in SCT, the videos address diabetes education on medication adherence, glucose monitoring, stress management, healthy eating and cooking, and strategies to increase physical activity. In total, 24 DSMES videos have been developed and previously tested in our pilot study, which demonstrates their feasibility and acceptability among Chinese immigrants with poorly controlled T2D [18–20].

The CHW support calls are informed by the NIMHD Research Framework, which provides a guidance for addressing community- and societal-level factors that influence diabetes outcomes [21]. To enhance diabetes knowledge and promote access to care, the CHW component complements the DSMES videos by identifying participants’ social needs (e.g., access to healthy food, medications, or blood sugar testing supplies), linking participants to community and clinical resources, assisting with healthcare navigation, and providing advocacy during clinical visits. Table 1 presents the detailed components of the LINK-IT intervention.

**Table 1:** The detailed components of the LINK-IT intervention.

Week	Video Topic	Key Contents of Each Video	CHW Supportive Calls
1	Diabetes Overview	Type 2 diabetes is a chronic disease characterized by high blood sugar. Poor control can lead to complications, including stroke, heart disease, kidney failure, amputation, and blindness. Although Diabetes cannot be cured, blood sugar can be managed through diet, exercise, medication, and glucose monitoring.	Reinforce the video education. Assist in goal setting. Address SDOH barriers. Provide support. (Biweekly throughout 24 weeks)
2	Life Goals	Maintaining blood sugar control requires ongoing motivation and self-discipline (e.g., family-centered motivation). Reflection prompts: “Do you want to control your blood sugar? What is your motivation?”	
3	Setting Goals	Set both long-term and short-term goals to boost motivation and confidence. Goals should be specific and actionable (e.g., add leafy greens to meals, walk 15 min tomorrow).	
4	Healthy Eating for Diabetes (Part 1)	Healthy eating is one of key strategies for blood glucose control. Dietary intake consists of three primary macronutrients: carbohydrates, protein, and fat. A balanced diabetes meal plan typically includes a plate composition of approximately 2 parts non-starchy vegetables, 1 part protein, and 1 part starch. Simple sugars and refined grains (e.g., baked sweets, sugary beverages, white rice, white bread) can cause rapid increases in blood glucose and should therefore be limited. Complex carbohydrates (e.g., whole grains, legumes, non-starchy vegetables) support more stable glycemic responses and are recommended as preferred carbohydrate sources. Proteins: Adequate protein intake helps maintain muscle mass and supports satiety, which contributes to more stable blood glucose levels. Choose lean proteins over processed meats. Prefer lean animal proteins (e.g., poultry, deep-sea fish) and plant-based proteins (e.g., soy milk, tofu, edamame). Limit red meat intake and select lean cuts when consumed. Avoid processed meats (e.g., bacon, sausage, deli meats) due to high sodium and saturated fat. Recommend consumption of deep-sea fish twice per week to support cardiovascular health.	
5	Healthy Eating for Diabetes (Part 2)	Healthy fats (e.g., plant oils, avocado, nuts/seeds, deep-sea fish) support blood glucose control and cardiovascular health. Unhealthy fats, including trans fats (e.g., baked/fried foods), high saturated fat foods (e.g., coconut oil, fatty meats, butter), reduce insulin sensitivity, raise cholesterol, impair glucose control. Cooking methods: prefer steaming, boiling, stewing, roasting, or light stir-frying; avoid deep frying and heavy sauces. Examples of Diabetes Plates.	
6	Grocery Shopping Guide	Vegetables/fruits: prioritize leafy greens and low-sugar fruits; limit starchy/high-sugar items. Protein: choose lean animal proteins (white meats) and legumes; limit red meats. Oils/fats: select olive oil or vegetable oil; avoid butter and lard. Diary: choose low-fat products. Grains: choose high-fiber whole grains; avoid instant or refined cereals. Beverages: choose sugar-free drinks; avoid sugary beverages. Shopping Tips: shop the store perimeter; read nutrition labels carefully.	
7	Diabetes Medication Management	Proper medication adherence is crucial for preventing complications. Strategies: use a pill organizer, place medications in visible locations, set reminders, involve family support. Do not adjust without consulting a clinician. Carry fast-acting sugar to treat hypoglycemia.	
8	Monitoring Your Blood Sugar	HbA1c measures the average blood glucose over 2–3-month (target <7%). Fingerstick monitoring provides real-time glucose levels. Check blood sugar before/after meals, snacks, exercise, medication, or stress. Use monitoring results to adjust diet, exercise, and medications.	
9	Exercise and Diabetes	Exercise improves blood sugar, blood pressure, lipids, and weight management. Aerobic exercise includes walking, running, swimming, cycling, and gardening. Aim for moderate-intensity exercise for at least 30 min a day, 5 days a week. Incorporate movement throughout the day in small intervals.	
10	Training Muscles Through Weightlifting	Perform strength training at least 2 days/week using resistance (e.g., milk jugs, canned goods, dumbbells). Exercises: (1) Toe Raises, (2) Bicep Curls, (3) Glute Bridge. Repeat each exercise 10 times, rest 1–2 min, perform a second set.	
11	Rewarding Yourself	Break goals into manageable steps and set reminders for exercise. Use positive rewards to reinforce healthy behaviors.	
12	Visit the Doctor	ABCs of diabetes care: A1c (<7%), blood pressure (<130/80), and cholesterol. Visit a clinician at least twice a year. Prepare questions about progress, treatment goals, and management strategies. Discuss any medication side effects with the clinician.	
13	Support from Friends and Family	Family/friends can provide emotional support and motivation. Guidance may be needed to ensure support does not interfere with diabetes management. Encourage clear and effective communication to enhance support.	
14	Problem Solving: Obstacles and Setbacks	4-step problem-solving method: (1) identify problems, (2) determine the cause, (3) brainstorm solutions, (4) choose a feasible plan. Apply these skills to overcome barriers related to diet and exercise.	
15	Triggers for Unhealthy Behaviors	Identify external triggers that make blood sugar control difficult (e.g., seeing sweets or cookies). Strategies: limit exposure or replace with healthier options (e.g., nuts) Reflect on challenging situations and plan strategies to improve.	
16	Emotional Eating	Apply the 4-step problem-solving method for emotional eating: (1) track eating patterns, (2) identify emotional triggers, (3) develop coping strategies, (4) Plan for future stress- or emotion-related eating situations.	
17	Food Addiction and Habits	Highly sweetened foods may stimulate the brain similarly to addictive substances. Apply the 4-step problem-solving method: recognize patterns, identify triggers, select strategies, and plan meals in advance.	
18	Stress and Diabetes	Excessive stress can raise blood sugar levels. Coping strategies: talking with family/friends, exercise, engage in physical activity, watching TV, practicing deep breathing or meditation. Involve family/friends in supporting diabetes care.	
19	Negative Self-Evaluation	Negative self-talk can impair blood sugar management. Build confidence by practicing positive thinking, acknowledging daily successes, and setting achievable goals.	
20	Holidays and Dining Out	Maintain routines for medication, diet, exercise, blood glucose monitoring, and stress management during holidays. Tips: eat slowly, choose healthier menu items, control portion sizes, and seek support from family/friends.	
21	Preventing High-Risk Situations	Identify high-risk situations (e.g., stress, busy schedules). Review and adjust goals as needed. Apply problem-solving skills to prevent relapses and plan ahead.	
22	Setbacks and Relapses	Setbacks are a normal part of lifestyle change. Review medications, diet, and exercise routines Identify barriers and seek support to restart progress.	
23	How to See a Doctor in the US	Where to seek care: private clinics, community health centers, hospitals, emergency rooms, or urgent care. Inquire about financial assistance programs and low-cost medications. Contact a primary care provider or diabetes specialist for guidance and questions.	
24	Summary of What We’ve Learned	Maintain motivation and apply strategies to overcome ongoing challenges. Reinforce goal setting and support systems. Continue using the 4-step problem-solving method to sustain healthy behaviors: track behavior, identify causes, plan solutions, create a plan for future situations.	

#### Intervention arms.

**Fig 2:**
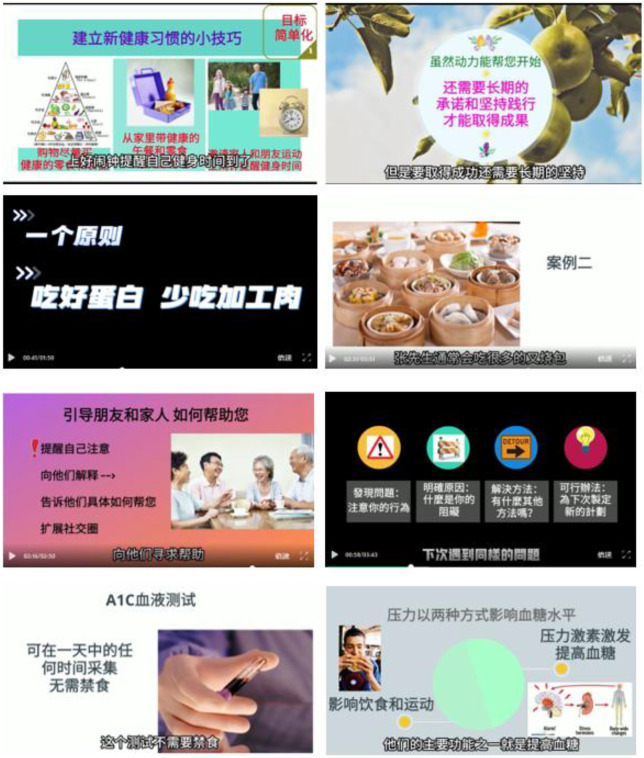
Example of the LINK-IT videos.

The LINK-IT trial employs a 3-arm RCT design:

1) **VIDEO+CHW**: Participants will receive 24 culturally and linguistically tailored DSMES videos delivered via text message links (one video per week for 24 weeks). Each video is approximately five minutes long and is grounded in SCT [28], emphasizing mastery experiences, social modelling, and skill-building for diabetes self-management (e.g., healthy cooking, medication adherence, and physical activity). Fig 2 presents examples of the video content. In addition, participants will receive biweekly telephone support from a trained, bilingual CHW throughout the 24-week intervention period. Each call will take approximately 10–30 minutes. The goals of the CHW calls are to: reinforce video content and support goal setting; systematically assess SDOH barriers (e.g., food insecurity, financial hardship for medications) using a structured form; link participants to community resources; and provide assistance with healthcare navigation and patient advocacy.

2) **VIDEO**: Participants will receive the same 24 DSMES videos (one video per week for 24 weeks) without CHW support.
3) **CONTROL**: Participants continue to receive usual medical care, allowing for estimation of the effect sizes of each intervention component relative to standard practice. Upon study completion, control participants are offered access to the full 24 DSMES videos.

This 3-arm design enables the trial to assess the efficacy of the video intervention alone, evaluate the additional benefit of personalized CHW support in addressing SDOH and barriers to care, and determine the overall effectiveness of the combined intervention compared to usual care. Importantly, it allows the study to quantify the incremental value of CHW support, understand mechanisms underlying improvements, and inform whether a digital-only approach could be sufficient for broad dissemination or whether additional community-level support is necessary to reduce health disparities.

#### CHW training and fidelity.

To ensure CHWs are well-prepared and maintain a standardized approach, they will complete a 35-hour initial training provided by the study team. The training covers core competencies including diabetes knowledge, culturally tailored diabetes self-management education, communication skills, motivational interviewing, goal setting, problem-solving strategies, identification and assessment of SDOH barriers, and available community resources. CHWs also receive guidance on patient advocacy and ethical considerations in research. Following the initial training, CHWs will receive ongoing supervision through weekly team meetings and participate in case discussions to address challenges, reinforce skills, and ensure adherence to the intervention protocol.

To monitor intervention fidelity, 10% of CHW audio-recorded calls will be randomly reviewed using a standardized checklist assessing protocol adherence, counseling quality, goal-setting support, and appropriate use of referral resources. Feedback from fidelity reviews will be provided to CHWs regularly to maintain high-quality and consistent delivery of the intervention.

### Outcomes

Outcomes will be measured at baseline, 6 months, and 12 months. Data will be collected via surveys administered by research staff over the phone.

#### Primary outcome.

**HbA1c level**: HbA1c will be assessed at baseline, 6 months, and 12 months. We will extract the EHR records to obtain enrolled participants’ HbA1c. In addition, given the missing data issues with EHR data, we will also use a point-of-care A1C test kit (*A1CNow® + system*) to collect participants’ HbA1c. This test kit has been widely used in diabetes trials and provides researchers with a fast, convenient, and reliable method for obtaining accurate HbA1c results from a simple fingerstick, capturing the most recent glycemic status [29,30]. Trained research staff will assist with HbA1c point-of-care measurements in a safe, private space at a community partner’s office, close to the participant’s home.

#### Secondary outcomes.

Secondary outcomes include:

1) **Self-Efficacy for Diabetes**: Self-efficacy refers to participants’ confidence in managing T2D. It will be measured using the Stanford Self-Efficacy for Diabetes Scale, a well-validated eight-item instrument that has been widely used in diabetes-related studies [31,32]. Participants rate their confidence in performing specific self-management behaviors on a 10-point Likert scale, ranging from 1 (not at all confident) to 10 (totally confident).
2) **Dietary Intake**: Dietary intake will be assessed using the Starting the Conversation Diet Scale [33], a brief, validated instrument designed to capture participants’ dietary behaviors. The scale assesses the frequency of key dietary practices, including fruit and vegetable consumption, sugar-sweetened beverage intake, and fast food consumption, providing a snapshot of overall diet quality. This tool has been widely used in studies related to obesity and diabetes across diverse populations, including immigrant groups [34,35].
3) **Physical Activity**: The short version of the International Physical Activity Questionnaire will be used to assess the frequency and duration of various physical activities over the past seven days [36]. This measure allows calculation of the total number of minutes per week spent in vigorous, moderate, and mild intensity activities [36], and is one of the most widely used tools to measure physical activities [37].
4) **Medication Adherence**: Adherence to prescribed medications will be assessed using the 11-item diabetes-specific version of the Adherence to Refills and Medications Scale [38], which captures behaviors such as missed doses, timing errors, and challenges in obtaining refills. Scores are calculated such that higher scores indicate poorer adherence [38].
5) **Emotional Support:** Emotional support will be assessed using the Emotional Support Subscale from the NIH Toolbox Adult Social Relationship Scales [39]. This validated instrument comprises eight items, which capture individuals’ perceptions of empathy and understanding received from others in their social network. Total scores range from 8 to 40, with higher scores reflecting greater perceived emotional support.

#### Covariates.

Sociodemographic characteristics, health status, acculturation, SDOH, and cognitive function will also be assessed at baseline. Basic sociodemographic and health information, including age, gender, marital status, education, employment, income, insurance, duration of residence in the U.S., English proficiency, diabetes medication regimen, duration of T2D, and relevant medical history, will be collected using a standardized sociodemographic sheet. Acculturation will be evaluated using the PINE Study Acculturation Scale [40]. SDOH will be assessed using the Accountable Health Communities Health-Related Social Needs Screening Tool [41], which allows for a comprehensive examination of community- and societal-level factors that may influence diabetes outcomes in this population. In addition, we will measure cognitive function using the Montreal Cognitive Assessment, a validated tool designed to assess multiple cognitive functions, including attention, memory, language, executive function, visuospatial skills, and orientation [42].

### Data management plan

All study data will be de-identified and managed in accordance with NYU Langone Health IRB protocol and federal guidelines to ensure privacy and data integrity. Electronic data, including survey responses, intervention metrics, and clinical outcomes, will be stored securely on NYU REDCap, with access limited to authorized personnel. Paper records will be stored in locked cabinets in the researcher’s office. Mobile device data, such as video engagement and CHW call logs, will be securely stored with identifiers kept separate in NYU OneDrive and in REDCap. De-identified datasets will be archived for future analyses, and any external data sharing will require principal investigator approval and formal data use agreements.

### Safety considerations

The LINK-IT trial involves minimal-risk behavioral interventions, including video-based DSMES and CHW support. All study procedures will be conducted in accordance with NYU Langone Health IRB protocol to ensure participant safety and privacy. Participants will be informed that the interventions are educational and supportive and do not replace medical care; they will be encouraged to contact their healthcare providers for any medical concerns. Any adverse events or unexpected issues related to participation will be documented and reported promptly to the research team and the IRB. CHWs will be trained to identify urgent health concerns during support calls and refer participants to appropriate medical care as needed.

### Statistical analysis

An intention-to-treat approach will be used for all analyses [43]. Primary analyses will employ linear mixed-effects models with fixed effects for time, group (VIDEO+CHW, VIDEO, CONTROL), and their interaction, and a random intercept for each participant. These models will test the hypothesis of differential changes in the primary and secondary outcomes across the three groups at 6 and 12 months. Pairwise comparisons between groups (VIDEO+CHW vs. VIDEO, VIDEO+CHW vs. CONTROL, and VIDEO vs. CONTROL) will be conducted, with adjustment for multiple comparisons using the Bonferroni method. Model covariates will include any baseline characteristics found to be imbalanced across groups. Sensitivity analyses will be conducted to assess the impact of missing data. All statistical analyses will be performed at a two-sided significance level of 0.05 using R software.

### Ethics and dissemination

This study protocol has been approved by the Institutional Review Board of the NYU Grossman School of Medicine (S23-01274). Written or verbal informed consent will be obtained from all participants. The option of verbal informed consent has been approved by the IRB. All data will be de-identified to protect participant confidentiality. Study results will be disseminated through peer-reviewed publications, presentations at scientific conferences, and community forums. The findings will provide critical evidence on scalable models for delivering DSMES to underserved immigrant populations.

### Study timeline

Recruitment for the LINK-IT trial commenced on December 29, 2024. As of the manuscript submission date, the trial remains in the active recruitment phase, and neither participant enrollment nor data collection has been completed. We anticipate that participant recruitment will be completed by March 31, 2027, data collection by March 31, 2028, and that study findings will be available by August 31, 2028. A detailed study timeline is provided in the Supporting Information File (S1 Table). The actual study progress is in line with the study timeline. The original trial protocol is presented in the Supporting Information file (S2 File).

## Discussion

This report presents the protocol for the LINK-IT Trial, a 12-month, three-arm randomized controlled trial evaluating a multilevel intervention that integrates a culturally tailored, video-based DSMES program delivered via text message with CHW support to improve diabetes outcomes among Chinese immigrants. By combining behavioral education with assistance in navigating SDOH, this intervention targets multiple levels of influence on diabetes management in this traditionally underserved population. To our knowledge, this is the first fully powered RCT to test the efficacy of a personalized behavioral intervention for T2D management among Chinese immigrants with poorly controlled T2D. This theory-informed, multicomponent approach has the potential to advance diabetes care, enhance self-management behaviors, and improve diabetes outcomes in an underserved population. If proven effective, the LINK-IT intervention will offer a scalable, low-cost, and sustainable strategy for delivering culturally and linguistically concordant DSMES programs to a high-risk, underserved immigrant population. Furthermore, it may serve as a replicable model for other minority and immigrant communities facing similar barriers to chronic disease management, contributing to improved health outcomes for all communities.

Potential challenges include recruitment and retention within a hard-to-reach immigrant population and ensuring technological accessibility. However, these risks can be mitigated by multiple recruitment strategies, the use of a highly accessible text message platform, frequent communication with participants, robust community partnerships, and successful pilot work [18–20], which together demonstrate the team’s capacity to achieve recruitment and retention targets.

### Strengths and limitations

Strengths of this trial include its rigorous, fully powered, 3-arm RCT design, which enables evaluation of the independent and combined effects of video-based DSMES and CHW support. The intervention is culturally and linguistically tailored to address the unique needs of Chinese immigrants with poorly controlled T2D, and it integrates behavioral education with CHW support for navigating SDOH, targeting multiple levels of influence on diabetes management. Finally, the use of a highly accessible text-message platform and strong community partnerships enhances feasibility, scalability, and potential for real-world implementation.

Potential limitations include limited generalizability beyond Chinese immigrants in urban U.S. settings, as findings may not extend to other immigrant groups or rural regions. The reliance on self-reported measures for some secondary outcomes, such as dietary intake, physical activity, and medication adherence, may introduce reporting bias. Finally, the 12-month follow-up period may be insufficient to capture long-term maintenance of behavioral changes and glycemic control.

### Dissemination plans

Findings from the LINK-IT trial will be disseminated through multiple channels to reach both academic and community audiences. Peer-reviewed publications and presentations at national and international conferences will communicate scientific results to researchers, clinicians, and policymakers. Summaries of study findings will also be shared with broader Chinese communities in culturally and linguistically appropriate formats, including newsletters, community events, and digital media. Data sharing with external collaborators will be conducted in accordance with institutional policies, data use agreements, and participant confidentiality protections. Additionally, insights from the trial may inform the development of scalable interventions to reduce diabetes disparities among Chinese immigrants and other underserved populations.

## Supporting information

S1 Table Study timeline for the LINK-IT trial (September 2023 to August 2028; 5 years in total). (PDF)

S2 File Original study protocol. (PDF)

S3 File Protocol checklist. (DOCX)
