# Supplementary material for: Leveraging videos and community health workers to address social determinants of health in immigrants (LINK-IT): Protocol for a randomized controlled trial
Source: PLoS One. 2026 Feb 2;21(2):e0341217. doi: 10.1371/journal.pone.0341217 (PMC12863526; doi:10.1371/journal.pone.0341217)
Supplement: S1 Table — (PDF) [file pone.0341217.s001.pdf]

S1 Table. Study timeline for the LINK-IT trial (September 2023 to August 2028; 5 years in total).

**Year**	2023	2024				2025				2026				2027				2028		
**Quarter**	4	1	2	3	4	1	2	3	4	1	2	3	4	1	2	3	4	1	2	3
Finalization of clinical protocol and consent forms																				
Completion of contracts/third party agreements																				
Finalization of the intervention																				
IRB approval																				
Convene steering committee and data safety and monitoring board																				
Completion of regulatory approvals																				
Hire and train study staff																				
Registration of clinical trial in ClinicalTrials.gov																				
Enrollment of first subject																				
Enrollment and randomization, 25%, of the projected population)																				
Enrollment and randomization, 50%, of the projected population)																				
Enrollment and randomization, 75%, of the projected population)																				
Enrollment and randomization, 100%, of the projected population)																				
Completion of data collection time-period																				
Data cleaning																				
Interim and final data analyses																				
Closeout plans including communication of study findings to participants, as applicable																				
Completion of final study report and publication of primary outcomes																				
Reporting of results on ClinicalTrials.gov																				
