# Supplementary material for: Leveraging videos and community health workers to address social determinants of health in immigrants (LINK-IT): Protocol for a randomized controlled trial
Source: PLoS One. 2026 Feb 2;21(2):e0341217. doi: 10.1371/journal.pone.0341217 (PMC12863526; doi:10.1371/journal.pone.0341217)
Supplement: S2 File — (PDF) [file pone.0341217.s002.pdf]

# S2 File: Original Study Protocol

## LINK-IT: Leveraging vIdeos and commuNity health worKers to address socIal determinants of health in Immigrants

**Principal Investigator:**

Lu Hu, PhD
NYU School of Medicine
Department of Population Health
180 Madison Ave
New York, NY 10016
646-501-3438
lu.hu@nyulangone.org

**Protocol Number:** s23-01274

**Version #:** **Version 06.13.2025**

## TABLE OF CONTENTS


1. Abstract ..... 3
2. Purpose of the Study and Background ..... 3
   2.1 Background ..... 3
   2.2 Aims ..... 4
3. METHODS and procedures ..... 5
   3.1 Study Design ..... 5
   3.2 Sample and Sites ..... 5
   3.3. Characteristics of the Research Population ..... 6
4. Procedures ..... 7
   4.1 Patient Recruitment ..... 7
   4.2 Patient Screening ..... 7
   4.3 Patient Consent ..... 8
   4.4 Baseline Measurement ..... 9
   4.5 Follow-up visits ..... 10
   4.6 Payments ..... 11
5. Risk/Benefit Assessment ..... 13
   5.1 Potential Risks ..... 13
   5.2 Benefits ..... 13
6. Confidentiality and Data Storage ..... 13
7. Data Analysis and Data Monitoring ..... 14
   7.1 Data Analysis ..... 14
   7.2 Data Monitoring ..... 15
8. Investigators Qualifications ..... 16
9. Study Records Retention and Data Sharing ..... 16
10. References ..... 17

# 1. ABSTRACT

This proposed R01 is in response to the PAR-21-081 Addressing Health Disparities among Immigrant Populations through Effective Interventions. Chinese immigrants are the second largest immigrant group in the U.S., who suffer disproportionately high type 2 diabetes (T2D) burden and have poor diabetes outcomes. Diabetes Self-Management Education and Support (DSMES) programs are evidence-based interventions that provide patients with important knowledge and skills to navigate diabetes self-management at home. However, numerous social determinants of health (SDOH) barriers limit the access of DSMES programs to Chinese immigrants, including high rates of poverty, limited English proficiency (LEP), and lack of access to care and insurance. Given the high T2D burden and rapid growth in the Chinese immigrant population, there is an urgent need for research to make DSMES accessible to this minority group. High text message use in Chinese immigrants suggests a promising mechanism for enhancing access to DSMES. Yet, this mobile health (mHealth) intervention alone is likely insufficient to address many SDOH barriers reported by LEP Chinese immigrants (e.g., lack of insurance, financial barriers). The community health worker (CHW) model has been shown to be effective for addressing SDOH barriers in health disparity populations. Guided by the NIMHD Research Framework, we argue that a mHealth intervention that is supplemented with CHW support is a novel model for enhancing access to DSMES in Chinese immigrants. Our pilot work demonstrates the feasibility, acceptability, and potential efficacy of a text message-delivered video-based DSMES intervention. The goal of this R01 is to examine the efficacy of the video-based DSMES (hereafter VIDEO) or the video-based DSMES+CHW (hereafter VIDEO+CHW) intervention compared with a wait-list control group (hereafter CONTROL) to improve glycemic control among Chinese immigrants with T2D in NYC. Participants will be randomized with equal allocation to one of the 3 groups. The VIDEO group will receive 1 DSMES brief video/week for 24 weeks delivered via text message. The VIDEO+CHW group will receive same DSMES videos plus bi-weekly support calls from a CHW for 24 weeks. The CHW will assess participants' SDOH barriers to T2D care and link them to available resources in the community. The CONTROL group will continue to receive their usual care and at the end of the study, they will receive DSMES videos. The primary outcome is HbA1c at 6 months. This study will provide critical information on whether it is efficacious to use an existing social media platform or social media plus CHW support to enhance access to DSMES. If either of the interventions is proven efficacious, this project can provide important data for future scaling of this intervention. This study may serve as a program model for chronic care in other high-risk immigrants such as LEP Hispanic immigrants who also bear a high T2D burden, face similar barriers to accessing DMSES programs, and frequently use social media (e.g., WhatsApp).

# 2. PURPOSE OF THE STUDY AND BACKGROUND

## *2.1 Background*

Asian Americans are the fastest growing minority group and projected to surpass Hispanic population growth, becoming the largest immigrant group in the US by year 2065.[1] As the largest Asian subgroup,[2] Chinese immigrants bear high type 2 diabetes (T2D) burden, particularly in New York City (NYC) where roughly 1 in every 2 has T2D or prediabetes.[3–5] Compared to non-Hispanic whites, Chinese immigrants with T2D have higher rates of poverty and limited English proficiency (LEP) and poorer diabetes outcomes.[6–9] <u>Given the substantial and growing Chinese immigrant population and high T2D burden, there is an urgent need for effective diabetes control interventions in this group.</u>[10,11]

Diabetes self-management education and support (DSMES) programs are evidence-based interventions that provide patients with important knowledge and skills to manage T2D at home.[12] However, many social determinants of health (SDOH) barriers limit the access of DSMES to Chinese immigrants, including high rates of LEP, poverty, being uninsured, and poor access to

care.[13–15] Furthermore, there is a significant shortage of cultural- and linguistic-concordant certified diabetes educators to deliver DSMES.[16] These barriers are further amplified by the fact that DSMES is often delivered via multiple in-person visits, which can be challenging for LEP Chinese immigrants who often have long working hours and limited sick time from work.[14,15,17,18]

High text message use in Chinese immigrants suggests a promising mechanism for enhancing access to DSMES.[19,20] A mobile health (mHealth) intervention that leverages social media may have unprecedented advantages for increasing access to DSMES and holds strong promise for scalability.[21–23] Yet, mHealth alone cannot address many SDOH barriers to care.[24] The NIMHD Research Framework[25] highlights the importance of addressing multi-level SDOH barriers in health disparity populations. The community health worker (CHW) model has demonstrated efficacy for addressing SDOH barriers and providing critical support to underserved LEP immigrants.[26,27] We argue that a mHealth intervention that is supplemented with CHW support is a novel model for enhancing access to DSMES in Chinese immigrants. Despite the wide usage of text message in LEP Chinese immigrants,[28–30] no studies have examined the efficacy of a social media-based DSMES or social media plus CHW model to make DSMES programs more accessible.

In our prior study,[31] we successfully developed 24 culturally and linguistically tailored DSMES videos. Video links were shared via the free and popular social media platform with 30 Chinese immigrants with T2D. The mean video watch rate was high (92%). In our one-arm feasibility pilot, we found HbA1c at 6 months significantly declined by 0.6% compared to baseline (95% CI: 0.3%-0.9%, p=0.001).[31] Chinese immigrants also reported they needed assistance with SDOH barriers to T2D (e.g., lack of insurance, financial barriers to accessing medication or glucose testing strips). Building upon these pilot data, the overall goal of **this R01 study** is to examine the efficacy of the video-based DSMES (hereafter VIDEO), or the video-based DSMES+CHW intervention (hereafter VIDEO+CHW) compared with a wait-list control group (hereafter CONTROL) to improve glycemic control among 405 Chinese immigrants with uncontrolled T2D in NYC. Participants will be randomized with equal allocation to one of the 3 groups. The VIDEO group will receive 1 DSMES brief video/week for 24 weeks delivered via text message. The VIDEO+CHW group will receive the same DSMES videos plus bi-weekly support calls from a CHW for 24 weeks. The CHW will assess participants' SDOH barriers to T2D care and link them to available resources in the community. The CONTROL group will continue to receive their usual care and at the end of the study, they will receive DSMES videos. Measurements will occur at baseline, 6, and 12 months.

### *2.2 Aims*

**Aim 1**: Examine the efficacy of the VIDEO and VIDEO+CHW interventions on HbA1c (primary outcome) at 6 months

Hypothesis: *We hypothesize that the VIDEO+CHW group will have the largest reduction in HbA1c, followed by the VIDEO group, and then the CONTROL group at 6 months.*

**Aim 2**: Examine the efficacy of the VIDEO and VIDEO+CHW interventions on patient-centered outcomes (secondary outcomes) at 6 months. Secondary outcomes include enhanced self-efficacy, adherence to diabetes self-management behaviors, healthy eating, physical activity and medication taking.

Hypothesis: *We hypothesize that the VIDEO+CHW group will have the greatest improvements in secondary outcomes, followed by the VIDEO group, and then the CONTROL group at 6 months.*

**Aim 3 (exploratory)**: To explore the long-term impact of the VIDEO and VIDEO+CHW interventions on HbA1c at 12 months

**Aim 4**: Examine the reach, adoption, fidelity, and maintenance of the VIDEO+CHW intervention within clinical and community settings, and use the implementation frameworks (RE-AIM, CFIR) to delineate contextual factors influencing implementation outcomes (Implementation Evaluation)

## 3. METHODS AND PROCEDURES

### *3.1 Study Design*

| Intervention Components (rows) and Groups (columns) | CONTROL | VIDEO | VIDEO+CHW |
|---|---|---|---|
| Standard of T2D care | X | X | X |
| 1 DSMES video/week for 24 weeks | | X | X |
| Bi-weekly phone calls from CHWs on addressing SDOH barriers for 24 weeks | | | X |

**Table 1. Intervention Components (rows) and Groups (columns)**

The proposed study is a 3-arm RCT of 12 months duration. Participants will be randomized with equal allocation to one of 3 arms (n=135 each): 1) wait-list control (hereafter CONTROL); 2) video-based DSMES intervention (hereafter VIDEO); or 3) video-based DSMES+CHW intervention (hereafter VIDEO+CHW). All of the groups will continue to receive standard of care treatment for their T2D. The VIDEO group will receive 1 brief DSMES video per week for a total of 24 weeks. The VIDEO+CHW group will receive the same DSMES videos plus bi-weekly support calls from a trained CHW for 24 weeks. The CONTROL group will continue to receive their standard of care for T2D during the study duration and at the end of the study, they will be provided the opportunity to receive DSMES videos.

Rationale for a 3-arm RCT: The VIDEO intervention, if efficacious, would be more scalable than the VIDEO+CHW intervention, warranting investigation of the VIDEO intervention alone. However, evidence suggests that health disparity populations, including underserved low-income LEP immigrants, may require additional support to help address community level and SDOH barriers to care (e.g., lack of insurance, financial hardship to cover medication or test strips cost)[27,32], which the VIDEO intervention alone is unlikely to address. According to the NIMHD Research Framework[25], patient health outcomes will depend on multiple levels of influence, including individual, interpersonal, community, and societal levels. Within each level, there are 5 domains to consider, ranging from biology, behavior to healthcare system.[25] VIDEO intervention will provide important diabetes education to participants and empower them to become an activated patient on the individual level. In order to reduce health disparities and improve health outcomes, interventions should also consider other levels of influence and address SDOH barriers in LEP Chinese immigrants.[25,27] Use of CHWs has been shown to be an effective model for addressing SDOH barriers in underserved immigrants.[33,34] Therefore, we will examine the incremental effect of CHW support on video-based DSMES to understand whether there are any added benefits. A CONTROL group allows us to estimate the effect sizes for each of the intervention components compared to usual care and account for some potential unmeasured confounding variables. Findings from this 3-arm RCT will provide critical data on highly scalable strategies to address access issues of DSMES programs in health disparity populations.

### *3.2 Sample and Sites*

We will recruit 405 Chinese immigrants with uncontrolled T2D, who are currently receiving care at the Charles B. Wang Community Health Center (CBWCHC), a federally qualified health center located in both Manhattan Chinatown and Queens Chinatown. In 2020, CBWCHC provided a total of more than 233,000 service visits to more than 52,000 underserved Asian Americans in Greater NYC. The majority of patients are Chinese, limited English proficient (89%), and have an

income at or below 200% of poverty (84%). CBWCHC was chosen as our main recruitment site for several reasons: 1) there are existing collaborations with this site; Dr. Hu has been working with CBWCHC since 2016; 2) Dr. Shimin Cao is the Section Chief of Internal Medicine at the CBWCHC Queens Chinatown location and has clinical privileges at this site. He has been and will continue to act as a clinical site champion, and provide access to potential participants; and 3) CBWCHC is one of the largest and leading community centers in NYC, having established a trusting relationship with the Chinese American community

CBWCHC will not be engaged in research activities and their role is to identify/refer patients and provide space for this study. All research related activities (e.g. recruitment, consenting, conducting study procedures) will be performed by NYU study staff. The letter of support has been uploaded in the attachment section.

To maximize recruitment efforts, we will also identify potential private practices or community based organizations in Chinatown areas and work with them to recruit potential participants. In addition, we will identify and recruit participants into the study from NYU Langone Health. Similar to the procedures at CBWCHC, all of the research related activities (e.g. recruitment, consenting, conducting study procedures) will be performed by NYU study staff. The role of NYU Langone Health, these private practices or community-based organizations is to help to identify/refer patients and/or provide space for this study.

**Sample Size Estimates:** Primary Outcome (HbA1c at 6 months): The primary aim is to examine the efficacy of the VIDEO and VIDEO+CHW interventions on HbA1c (primary outcome) at 6 months. We hypothesize that the VIDEO+CHW group will have the largest reduction in HbA1c, followed by the VIDEO group and then the CONTROL group at 6 months (HbA1cVIDEO+CHW < HbA1cVIDEO < HbA1cCONTROL). Based on our preliminary data, we expect to observe a 0.6% difference between the VIDEO and CONTROL groups. Based on data of a prior CHW intervention in Hispanic immigrants with T2D, we expect to see a group difference of 0.4% between the VIDEO+CHW and VIDEO groups. Using the observed SD=0.8% of HbA1c in our preliminary study, based on a two-sample two-sided t-test, n=86 (per group) will be required to detect a minimum group difference of 0.4% in HbA1c with a power of 80% and a type I error of 0.0167 (to account for 3 group comparisons). We will abstract HbA1c data from the electronic medical record, which often has a high rate of missing data due to various reasons (e.g., patients miss the A1c appointment or change to a different health care system). Our pilot study achieved 70% completion rate of A1c follow up data. With a conservative rate of 60-65%, we will recruit 405 (135/group) participants to yield a final sample of at least 258 (86/group). We believe a sample of 405 will provide sufficient power to detect significant differences between the three groups (VIDEO+CHW vs. VIDEO; VIDEO vs. CONTROL; VIDEO+CHW vs. CONTROL).

### *3.3. Characteristics of the Research Population*

To be eligible for the study, participants must: 1) self-identify as a Chinese immigrant or Chinese American; 2) be above 18 years old, 3) have a diagnosis of T2D in the medical record; 4) have had an appointment with a physician for routine T2D care within the past 12 months; 5) have a most recent HbA1c ≥ 7%; 6) be willing to receive brief videos regarding T2D management, and 7) possess a smartphone or, if they do not have one, be willing and able to use a study smartphone. Individuals will be excluded from participation if they meet any of the following: (1) unable or unwilling to provide informed consent; (2) unable to participate meaningfully in the intervention (e.g., uncorrected sight and hearing impairment); (3) unwilling to accept randomization assignment; (4) is pregnant, plans to become pregnant in the next 6 months, or becomes pregnant during the study, or (5) is breastfeeding (e.g., they may have potential dietary restrictions).

## 4. PROCEDURES

### *4.1 Patient Recruitment*

Participants will be identified and recruited into the study from NYC health care facilities, including CBWCHC, private practices, community-based organizations, and NYU Langone Health and affiliated provider practices.

We will recruit participants using the following methods:

**a. Posters.** Posters will be placed in NYC health care facilities waiting and examination rooms. Posters will list a contact telephone number that patients can call if interested in enrolling. Posters will also be placed in Chinese community centers (e.g., senior centers) and distributed during Chinese community events.

**b. Direct referral by health care providers.** CBWCHC, NYU Langone Brooklyn site providers, and private practice health care providers will approach patients who are potentially eligible for the study, solicit their interest in the study. If patients express verbal interest in the study participation, their health care providers will share the patient's contact information with the study staff, or patients will also be able to call (self-refer to) to the number provided in the poster and speak with a study staff if they are interested in participating. Dr. Qiuqu Zhao is the medical director at NYU Brooklyn Family Health Center. We are collaborating with Dr. Zhao and her team at NYU Brooklyn. They would help refer potential study participants that are eligible and interested in our study. The initial referral list from health care providers will only include patient's name and phone number for those verbally expressed interest in the study. After verbal consent is obtained and documented, the study staff will request the patient's medical information from the medical record.

c. Electronic Medical Record Search. We will work with DataCore to generate a report to identify potentially eligible subjects across NYU Langone Manhattan, Brooklyn, and Long Island campuses. Using a query, search will be conducted in EPIC to identify Chinese patients aged 18+ and with type 2 diabetes whose most recent HbA1c value ≥ 7%. The report will also include demographics including patient name, gender/sex, date of birth, address, phone number, weight, height, BMI, name of primary care physician, most recent HbA1c value and date of measurement. Only the principal investigator and all members of the research team will have access to this report. The report will be generated prior to the recruitment process to help us find potential participants. The report will be kept on a secure electronic NYU research drive to ensure the confidentiality of health information and identifying information from potential participants. These patients will receive a provider letter which will be mailed to them, describing the study and letting them know how they can opt out of further contact. We will permanently delete the report once we meet our recruitment goal. We will replicate similar approaches with CBWCHC and other health care providers, who will perform an electronic medical record search to generate a list of potentially eligible patients at their sites. These patients will receive a provider letter which will be mailed to them, describing the study and letting them know how they can opt out of further contact.

### *4.2 Patient Screening*

Upon receiving the referral list (interested patient's name and phone number) from the providers listed above, the NYU bilingual study staff will call the patient and provide more details about the study. If patients expressed interest in participation, the study staff will administer several screening questions over the phone to screen whether or not the participant is eligible (see Telephone Screener).

For patients identified from EPIC and DataCore and electronic medical record search, we will wait 1-2 weeks after the provider letter is mailed out and then call the patients to provide more details about the study. If patients expressed interest in participation, the study staff will administer several screening questions over the phone to screen whether or not the participant is eligible (see Telephone Screener).

Once eligibility is confirmed, the bilingual study staff will send patients via text message a copy of the key information sheet and the verbal consent script schedule a follow up phone call to obtain verbal consent. For those who screened ineligible, we will remove identifiers from the screening data and keep the de-identified data to generate CONSORT diagram for our final manuscript. This is necessary because in the final manuscript, as a researcher, we need to report the recruitment numbers (how many we called, how many we screened ineligible and ineligible reasons). For such purposes, we need to keep de-identified screening data to generate this CONSORT diagram.

### *4.3 __Patient Consent__*

Written Consent:

If the participant prefers to provide consent during an in-person visit, we will schedule an in-person visit. A comprehensive written consent form (available in English and Chinese) will be provided to the potential participant. This form will include information about the study purpose, study procedures, potential benefits and risks, data confidentiality, and the participant's rights, including the right to withdraw at any time without any negative repercussions. Participants will be given adequate time to review the consent form before making a decision. They will be encouraged to ask questions and ensure that they fully understand all aspects of the study before signing the consent form. For those who provide written consent, we will measure their weight and height and obtain their demographic information.

Verbal Consent:

The NYU study staff will make a second phone call to the participant by reading the approved telephone verbal consent. The verbal consent is an accommodation due to the ongoing COVID-19 pandemic in New York, and that the target population for this study is low-income aging immigrants with limited education and low digital literacy. All interventions will be conducted via phone or text message. A waiver of documentation of consent has been requested and approved.

The study team has requested a waiver of signed HIPAA authorization and all HIPAA information will be presented verbally. Under the HIPAA section, we will explain the potential risks of text message. Participants can decide on their own whether they would like to continue to participate.

An IRB-approved verbal consent script will be used and will include the HIPAA information, the subject will verbalize comprehension of the consent form and study enrollment. The date of consent will be documented on the Enrolled Participants Log. The Enrolled Participants Log is the only record linking a participant’s name and Subject ID, this information will be kept on a secure electronic drive to ensure the confidentiality of health information and identifying information from participants. Only the principal investigator and all members of the research team will have access to this information.

We will have the verbal consent script available in both English and Chinese. The participant can choose their own preferred language and our bilingual study staff will explain the

study to the participant. The participant will be encouraged to ask any questions they may have before providing the verbal consent.

After verbal consent is obtained, we will schedule third phone call to start baseline study questionnaire survey.

### *4.4 Baseline Measurement*

During the third phone call, participants will complete a baseline battery of self-report questionnaires. The bilingual study staff will facilitate these assessments and will provide instructions and/or assistance as needed. These phone calls will be audio recorded for data integrity and quality assurance. We will randomly select phone call audio recordings to ensure that they are following our study protocol. Audio recordings will be password protected and stored on the NYU Langone shared drive which is only accessible to our study team members. We will destroy the recordings in July 2029, one year after our grant ends.

#### *4.4.1 Randomization*

The study biostatistician will create a prior randomization sequence using a computer-generated list of random numbers. The independent study staff will inform participants of their group assignment and explain the next step after finishing questionnaires during the baseline phone call.

#### *4.4.2 Intervention Arms*

**Wait-list control group (CONTROL):** Participants in this group will continue to receive the standard of usual care for their T2D at their doctor's office during the course of our study. At the end of the study, the CONTROL group will be provided the opportunity to receive DSMES videos.

**Video-based DSMES (VIDEO):** Participants in the VIDEO group will receive brief pre-recorded DSMES videos, which include both educational and SCT-based behavioral content. All intervention videos have been linguistically and culturally tailored and pilot tested in a prior study.[31] Similar to the prior study, we will deliver video links via text messages. We will send 1 DSMES video each week for a total of 24 weeks with each video lasting about 5 minutes in duration. Our preliminary data demonstrated high feasibility and acceptability of these DSMES videos.

**Video-based DSMES+CHW (VIDEO+CHW):** Participants in this group will receive the same DSMES videos as described above. In addition, they will also receive brief phone calls from CHWs every 2 weeks during the 24 weeks video program. During these calls, CHWs will assess whether participants need assistance on SDOH barriers (e.g., health insurance enrollment, financial assistance on covering medication/test strips, food assistance). If needed, CHWs will link participants to available services within the community. CHWs will also provide assistance on navigating the complex health care systems and serve as an advocate for patients during doctor visits if needed. These phone calls will be audio recorded for data integrity and quality assurance. We will randomly select phone call audio recordings to ensure that they are following our study protocol. Audio recordings will be password protected and stored on the NYU Langone shared drive which is only accessible to our study team members. We will destroy the recordings in July 2029, one year after our grant ends.

### *4.5 Follow-up visits*

The study will also include 2 follow-up phone calls: 6, and 12 months. Survey data will be collected over the phone. These phone calls will be audio recorded for data integrity and quality assurance. We will randomly select phone call audio recordings to ensure that they are following our study protocol. Audio recordings will be password protected and stored on the NYU Langone shared drive which is only accessible to our study team members. We will destroy the recordings in July 2029, one year after our grant ends. We will abstract the HbA1c testing results from the medical record at the participant's doctors' offices. Given the missing data issue of the EHR record, we will also use the A1CNow Self Check Device (PTS Diagnostics, Whitestown, IN) to collect participants' most recent HbA1c value. The A1CNow Self Check Device is only utilized for research purposes and may not be used or relied upon as a diagnostic tool. Any concerning results will be shared immediately with their healthcare provider if the healthcare provider is unaware. Our research team has no affiliation with PTS Diagnostics.

**Study Variables**

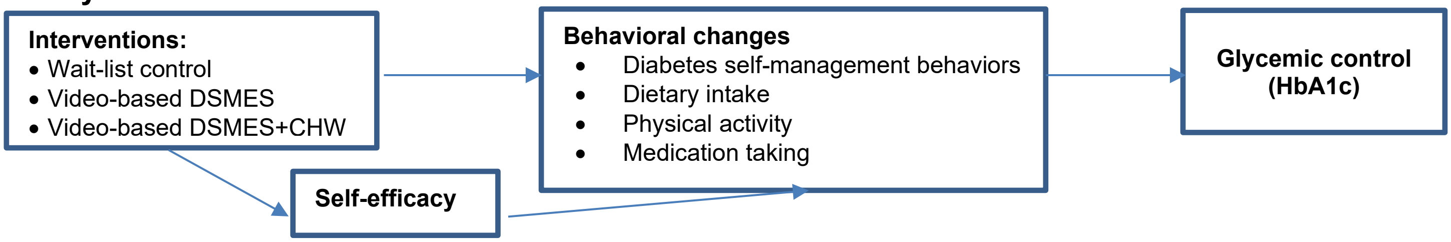


**Figure1. Study Variables**

**Primary & Secondary Outcomes Primary outcome: HbA1c:** As part of the usual care, patients with T2D receive a HbA1c blood test at their doctors' office every 3-6 months. we will abstract the HbA1c testing results from the medical record at the participant's health care facility. Once we obtain the consent from participants, we will contact their doctors' offices for HbA1c results. Given the missing data issue of the EHR record, we will also use the A1CNow Self Check Device (PTS Diagnostics, Whitestown, IN) to collect participants' most recent HbA1c value. The procedures are similar to the blood glucose finger prick that participants perform at home. Our trained study staff will guide the participants during this process. The A1CNow Self Check Device is only utilized for research purposes and may not be used or relied upon as a diagnostic tool.

**Secondary outcomes:** Consistent with DSMES programs (Figure 1), we will measure self-efficacy, diabetes self-management behaviors, dietary intake, physical activities, and medication taking at each time points (baseline, 6-, 12-months). All of these surveys have been used and validated in our prior and current studies with Chinese immigrants[31,35,3]>.

1. **Self-efficacy:** We will use the well-validated Stanford Self-Efficacy for Diabetes Scale[3]> to measure participants' confidence to manage T2D. This instrument contains 8 items and asks participants to rate their confidence level in performing specific self-management behaviors, using 10-point Likert scale ranging from 1 (not at all confident) to 10 (totally confident). This

instrument has been widely tested and has demonstrated excellent psychometric properties in Chinese immigrants.[38–40]

2. **Diabetes Self-Management Behaviors:** We will use the Summary of Diabetes Self-Care Activities (SDSCA)[4]> to assess participants' adherence to diabetes self-management behaviors. This scale consists of 11 core items and asks participants to describe adherence to diabetes self-care activities over the past 7 days. The reliability and validity of this scale has been well established in Chinese immigrants.[36,42]
3. **Dietary Intake:** Informed by a previous study and our own studies in Chinese Americans,[29,3]> we will use the 8-item Starting the Conversation diet scale[43] to estimate dietary intake behaviors in our participants.
4. **Physical Activity:** We will use the International Physical Activity Questionnaire (IPAQ)[45,4]> short version to assess the frequency and duration of various physical activities undertaken by adults over the past 7 days. This scale will provide an estimate of the number of minutes per week participants engage in each category of physical activity (e.g., vigorous, moderate, and mild intensity).
5. **Medication Taking**: We will use the Adherence to Refills and Medications Scale (ARMS) to assess adherence to medication-taking at baseline, 6, and 12 months.

**Covariates: Sociodemographic, health characteristics, acculturation, and SDOH.** We will use a sociodemographic and health questionnaire to collect basic information about the participant such as age, gender, education, income, duration of residence in the US, English proficiency, diabetes medication regimen, duration of T2D, and medical history. We will also use the PINE Study Acculturation Scale[4]> to evaluate various levels of acculturation in the sample and adjust for this in the statistical models if needed. This scale has established reliability and validity in Chinese immigrants. We will use the Social Determinants of Health (Core) survey[48] from the NIH PhenX Toolkit to assess SDOH in Chinese immigrants.

### *4.6 Payments*

The study will include 3 telephone surveys, baseline, 6, and 12 months. Patients will be paid $30 by Clincard which will be mailed to them for each survey. If participants finished all three surveys, they will receive a total of $90 in Clincard. We will also provide patients with an additional $20 at baseline, 6 months, and 12 months for completing the A1CNow Self Check test, totaling $60. In total, patients will receive up to $150 for completing the study.

### Aim 4 Implementation Evaluation

**4.7 Evaluation Framework.** We will utilize the RE-AIM framework to evaluate the impact of the proposed intervention. We will also use CFIR framework to evaluate facilitators and barriers to the implementation process (See **Table 2**).

**Table 2: RE-AIM and CFIR Evaluation Frameworks**

| CONSTRUCTS | MEASURES (Quantitative/Qualitative) | DATA SOURCES | TIME FRAME |
|---|---|---|---|
| **REACH:** What % of the target population came into contact with the program? Were participants representative of the target population? | | | |
| Participants | • % patients with uncontrolled T2D<br>• % patients at risk who received the VIDEO+CHW intervention<br>• Representativeness of patients referred and participated in study | • EHR data<br>• In-depth interviews with providers and CHWs | • Baseline, 6 months, 12 months |
| **EFFECTIVENESS:** Did program achieve key targeted outcomes? Did it produce any unintended adverse consequences? | | | |

| | | | |
|---|---|---|---|
| Primary Outcome | • Change in HbA1c at 6 months | • Patient data from EHR | • Baseline, 6 months, 12 months |
| Secondary Outcomes | • Change in HbA1c at 12 months and changes in self-efficacy,[140] diabetes self-management behaviors,[141,142] dietary intake,[20,143,144] physical activity,[20,145] medication adherence,[146,147] and SDOH[148] (at 6 and 12 months) | • Patient data from EHR<br>• Patient survey<br>• Healthify referrals | • Baseline, 6 months, 12 months |
| **ADOPTION**: Did the organization use the program? | | | |
| Adoption | • Utilization patterns | a) Provider, CBO, CHW interviews<br>b) Utilization reports (Redcap)<br>c) % of DSMES videos being watched; # of CHWs calls and duration of the call (Redcap) | a) Baseline, 12 months<br>b,c) Weekly |
| **IMPLEMENTATION (CFIR constructs to systematically guide identifying barriers/facilitators to implementation)**: How closely did staff members follow the program (consistency of delivery)? How well did the staff adhere to intervention fidelity? Was the program delivered as intended? Was the program consistent and aligned with clinic sites' missions? | | | |
| **CFIR** – Intervention Characteristics/<br><br>Fidelity | a) % of staff attending trainings and orientations; utilization patterns<br>b) # DSMES videos watched by the patient<br>c) # CHW phone calls attended per patient and fidelity to the protocol<br>d) Utilization patterns of CHW case notes | a) Training log, trainee evaluations; Utilization reports (Redcap)<br>b) Video watch records (Redcap)<br>c) Fidelity checklist, CHW encounter logs (Redcap)<br>d) Redcap utilization records; | a) Baseline; Post training; 6 months<br>b,c) Monthly<br>d) Weekly |
| **CFIR** – Characteristics of Individuals | • Attitudes, norms, self-efficacy, and intention[151,152] | • Provider surveys | • Baseline, 12 months |
| **CFIR** – Inner Setting | • Barriers and facilitators to workflow and referral processes | • Provider, CBO, CHW interviews/surveys | • 12 months |
| **CFIR** – Outer Setting | • Perceived contextual barriers and facilitators | • Provider, CBO, CHW interviews | • 12 months |
| **MAINTENANCE**: Is the organization willing to sustain the program? Is the program able to become part of routine practice? | | | |
| Practice patterns | • Current site patterns, barriers and facilitators | • Provider, CBO, and CHW interviews | • 12 months |
| Organizational characteristics | • # patients, FTEs, staff characteristics | • Baseline surveys with sites | • Baseline |

**D4.2. Data Sources and Collection:** 1) To capture data on *utilization patterns* of the intervention, we will extract system files from the RedCap databases every week that have date and time stamps and user logins to assess participation and completion of intervention components. Data will be extracted and analyzed by the NYU research team. 2) At baseline and follow-up, study coordinator will administer *brief surveys* to participating physicians (n=10) to capture data on: satisfaction with workflow before and after intervention, information sources before and after intervention, acceptability of and satisfaction with the integrated intervention, and barriers and facilitators of care coordination tools, building on our prior work.[116,120,125,126] 3) Study coordinator will conduct *qualitative key informant interviews* with physician champion and/or administrators at CBWCHC, community organizations that serve as referral sites, and CHWs (n=15). At baseline, interviews will assess current satisfaction and usage CHW and organizational workflows. At follow-up, the interviews will assess barriers and facilitators to the implementation and adoption process of the integrated intervention, appropriateness, fidelity to the interventions, and to solicit recommendations for the replication and scalability of the intervention to other sites. We will also assess satisfaction and outcomes from the partnership and capacity building process. Questions will be adapted from existing validated measures on acceptability, feasibility, adoption, organizational culture, and scalability and our previous work.[153,154] 4) We will conduct *fidelity checklists* during CHW biweekly phone calls for the first 2 months to assess that CHWs are covering all the key points outlined in the protocol. A kappa fidelity score will be calculated based on the percentage of key points completed, and provided to the study team. If CHW cannot improve fidelity to > 80% after two months, he/she will not be retained. In addition, CHWs will

keep encounter logs of all interaction with study participants and materials will be reviewed monthly by the study coordinator.

## 5. RISK/BENEFIT ASSESSMENT

### *5.1 Potential Risks*

Risks of participation in this study are minimal and unlikely to occur given the safeguards in place. Psychological risks include: 1) the possibility that some participants may perceive some questionnaire or background data assessment as intrusive or causing them to feel uncomfortable; 2) breach of confidentiality. The study involves an intervention designed to enhance adherence to standard diabetes care (e.g., adherence to the prescribed diabetes medication, healthy eating, and physical activity behaviors). If our intervention improves adherence to the medication regimen or if participants make lifestyle changes (e.g., increased physical activity), more episodes of blood sugar fluctuation may result. We will advise participants of the importance of monitoring their blood sugar as suggested by their doctors, and to contact their doctors as soon as they experience low blood sugar (<70mg/dl) or high blood sugar (based on the level advised by their doctors, as the upper limit varies between patients/providers). We will report these episodes of low and high blood sugar to Medical Monitor within 2 business days of knowing these events.

This study involves the use of text messages to deliver diabetes video links to participants. There are some security concerns with regard to text messages. We acknowledge these concerns, however, we think the benefits outweigh the risks. These low-income Chinese immigrants are already familiar with using text messaging and it is part of their daily life. Requiring them to download a new app or using a new portal would be extremely challenging due to the limited digital literacy issues in this population. We have used text messages in research studies over the past 5 years and have not encountered any privacy or confidentiality concerns. We have and will continue to work closely with our Medical Center IT department to ensure patient privacy and confidentiality. We will replicate measures used in our prior studies including: 1) assigning a unique code to each participant for accessing the intervention videos; 2) directing participants to refrain from sending any PHI via text messages and to call the study phone number for any questions; 3) study staff will use NYU encrypted study phones, that are HIPAA compliant; 4) all study staff will be trained on how to communicate with patients with regard to text messages.

### *5.2 Benefits*

Participants may benefit from the DSMES as they may decide to exercise more, make healthy dietary choices, and other lifestyle modifications that may lead to improved diabetes management. Information learned during the study may also produce benefits for future patients by improving their diabetes care.

## 6. CONFIDENTIALITY AND DATA STORAGE

We will use the IRB recommended RedCap for data storage (openREDCap.nyumc.org). We will also work with MCIT to set up a NYU shared network drive which is HIPAA compliant (for offsite backup storage) specifically for our study. All study personnel have already taken the mandatory HIPAA and Patient Privacy/Confidentiality training modules required by our institutional IRBs, to ensure that they are aware of the importance of patient confidentiality and all appropriate laws

regarding protection against privacy breaches. Procedures will be in place to ensure that all files containing subject information will be kept in locked filing cabinets or password-protected electronic databases on a secure server. The majority of study data will be maintained in electronic files with no patient identifiers other than a study ID number; there will be one file maintained separately with its own password protection that links study ID numbers to patient identifying information.

No identifiable information about study participants will be disclosed to individuals outside those approved by the IRB to be on the study protocol. No publications that result from the study will identify individual participants.

The text-message-based video counseling will be protected via password-enabled access. Only study participants will have access codes to open these videos. Others in the same household would not be able to open the video, unless participants choose to share the access code.

## 7. DATA ANALYSIS AND DATA MONITORING

### *7.1 Data Analysis*

**Analytic Plan for Aim 1**: An “intent-to-treat” approach will be used to address the specific aims. For Aim 1, we hypothesize that the VIDEO+CHW group will have the largest reduction in HbA1c at 6 months, followed by the VIDEO group, and then the CONTROL group. A random effects linear mixed modeling will be used to test time-specific differences attributable to the intervention. We also will use the “lincom” command in Stata to estimate differences in time-specific changes from baseline. Randomization should obviate the need for adjustment, but in the case of unbalanced baseline covariates, these will be included as necessary in adjusted analyses. We will use splined linear mixed models with repeated measures to compare changing trends in different periods: 0-6 months and 6-12 months. In this analysis, we will adjust for the covariates as needed.

**Analytic Plan for Aim 2**: For Aim 2, we hypothesize that the VIDEO+CHW group will have the greatest improvements in secondary patient-centered outcomes. Similar to the plan for Aim 1, we will use random effects linear mixed models, adjusting for relevant covariates in the case of unbalanced covariates between the treatment arms at baseline. We will use splined linear mixed models with repeated measures to compare changing trends in different periods, adjusting for the covariates as needed.

**Analytic Plan for Aim 3**: For exploratory Aim 3, we hypothesize that the VIDEO+CHW group will have the largest reduction in HbA1c at 12 months. Similar to the plan for Aim 1, we will use linear mixed effects models and splined linear mixed models with repeated measures to compare changing trends in different periods, adjusting for relevant covariates if needed.

**Analytic Plan for Aim 4.** Utilizing the RE-AIM framework, we will address “reach” by comparing demographic and clinical characteristics of patients enrolled vs. eligible and not enrolled, using Wilcoxon’s rank-sum test or Fisher’s exact test. We will also examine these factors separately by sex and age brackets. To address “effectiveness”, we will use the random linear mixed modeling to examine whether the VIDEO+CHW intervention group had a greater reduction than the CONTROL group. For “adoption”, we will use Chi-square tests to compare the proportion of patients completing intervention components. To address “implementation”, we will report the frequency of barriers/facilitators to implementation, as well as other fidelity measures, and Chi-

square tests to compare proportions. Finally, both “implementation” and “maintenance” will be explored qualitatively as described below.

Barriers and facilitators to implementation of the VIDEO+CHW intervention. All interviews will be audio-recorded and transcribed. We will use "constant comparison" analytic approach. The “constant comparison” approach is a method of explanation building in which the findings of an initial case are compared to a provisional category, revised as necessary and then other details or new cases are then compared against the revision and revised again as needed. This process is continued until an area of interest is fully explicated, reaching theoretical saturation. Using "thematic" coding, we will develop an initial set of codes, which will be reviewed by the Steering Committee to ensure they are relevant and complete. For each core code, we will ultimately develop one or more "secondary codes" that represent either more specific or restricted aspects of the phenomenon, to contextualize it, or to suggest underlying meanings. The secondary codes will vary in specificity or subtlety depending on the judged substantive value of additional refinements. Transcripts will be coded by at least four coders (graduate and post-doctoral students). Discrepancies in coding will be discussed and resolved, then the process is repeated with a new set of transcripts until an acceptable level of inter-coder reliability between them has been by the achieved, estimated using an appropriate chance-corrected statistic (e.g., kappa for nominal data and T-index for ordinal data). We will use Atlas.ti software to analyze the transcripts.

### *7.2 Data Monitoring*

The purpose of the data safety monitoring plan is to ensure the safety of subjects and the validity and integrity of the data. Data and safety monitoring will be the shared responsibility of all members of the research team. Personnel involved in monitoring activities will include:

- Dr. Lu Hu, PhD, PI, Dr. Hu has been involving in multiple clinical trial studies and she has beenactively participating in Dr. Sevick’s project meetings, including data and safety monitoring meetings. Dr. Hu has received intensive training in human subjects research. Dr. Hu will be responsible for data safety monitoring of the overall study and Dr. Sevick will provide close oversight via weekly mentoring meeting.
- Dr. Olugenba Ogedegbe, MD, MPH (sub-investigator). Director of the Institute for Excellence in Health Equity, Professor of Population Health at NYUSoM. Dr. Ogedegbe who has medical training and clinical experience will serve as the medical monitor, review and follow adverse events and oversee the safety of the study.

**Data monitoring**

Ongoing quality control will include regular data verification and protocol compliance checks. An ongoing review of study procedures will be done to ensure that the privacy of subjects and confidentiality of data is not violated. Weekly meetings will be held between Dr. Hu and Dr. Sevick and other research staff involved in the project to review the progress of subjects enrolled in the study.

**Safety monitoring**

It is possible that our intervention might help participants better adhere to the medication regimen or make lifestyle changes (e.g., increased physical activity), which may result in the need for less diabetes medications and thus more episodes of hypoglycemia might happen. We will advise them to continue to monitor their blood sugar levels as suggested by their doctors, and contact their doctors if they experience any low blood sugar levels. Our study staff will

emphasize this safety information during each follow up phone calls. Dr. Olugenga Ogedegbe, MD will serve as the Medical Monitor of the study and oversee the safety of the study.

Because the proposed study is minimal risk, we will not establish an independent Data Safety and Monitoring Board. The study team including several experts (Drs. Sevick, Hu) who have extensive experience in conducting clinical trials will perform date safety and monitoring. We will meet regularly to review inclusion and exclusion criteria, and develop a detailed safety protocol, plans for monitoring subjects for adverse events, and a protocol for protection of privacy and confidentiality of participants. The study PI (Dr. Hu) will hold weekly project meeting with the study staff and provide project updates to Dr. Sevick (primary mentor) via weekly mentoring one-on-one meetings. The study PI will provide updates on the study progress, recruitment, retention, data quality, safety, confidentiality, and the occurrence of adverse events to the whole mentoring team during bi-monthly check-in meetings.

<u>The whole team will provide oversight for the following study procedures:</u>

- Monitoring the safety of the subjects (e.g., review the research protocol and plans for data and safety monitoring)
- Evaluate the progress of the clinical trial, including periodic assessments of data quality and timeliness, subject recruitment, accrual and retention, subjects risk versus benefit, performance of the recruitment site, and other factors that can affect study outcome
- Maintaining the confidentiality and integrity of the data
- Reports of critical or adverse events from research staff. The PI will receive these reports on an event-by-event basis and will inform the whole study team of all such reports
- Make recommendations to the IRB and investigators concerning continuation or conclusion of the trial.

## 8. INVESTIGATORS QUALIFICATIONS

Lu Hu, PhD, is well poised to serve as the PI on the proposed study based on 1) her rigorous doctoral training in chronic disease management and postdoctoral and 2016 NIMHD Summer training in health disparity, 2) her current role as a PI on several diabetes studies in Chinese immigrants and as a co-investigator on an Aetna-funded study involving the use of a mHealth intervention (e.g., text messages) to remotely titrate the insulin regimen in underserved low-income patients with uncontrolled type 2 diabetes (T2D), 3) a strong publication record of about 32 published/accepted papers, and 4) several national and international awards and fellowships.

Olugbenga Ogedegbe, MD, MPH is a Professor at NYU School of Medicine. His work focuses on the development, implementation, and translation of evidence-based interventions targeted at cardiovascular risk reduction in minority and low-income populations at practice-based settings. He has clinical and medical expertise and will serve as the Medical Monitor for this study.

All investigator’s CVs are attached to the protocol submission.
All investigators and staff involved in this project have completed training on the protection of human subjects in research through CITI training and HIPAA certification.

## 9. STUDY RECORDS RETENTION AND DATA SHARING

We are firmly committed to sharing data with the scientific community so that the data generated from this study can be fully utilized for research. Provided below is the proposed data-sharing plan:

1. We will publish the detailed methodology used for this study. Aggregated statistics will be provided to the broad scientific community via a journal's website or to an individual investigator/team upon request.

2. We will release the data generated from all study participants to qualified researchers who wish to collaborate with the investigators from our study. Investigators who wish to collaborate should submit a proposal that will be reviewed using criteria similar to those used by the NIH \for scientific merit and human subject protection. All data should be used for research only. No data will be provided that could potentially disclose the identities of study participants.

NIH may release new policies regarding data sharing during the study period. We will be willing to discuss such policies with NIMHD program officers and modify the data-sharing plan detailed above.

## 10. REFERENCES

1. Budiman A, Ruiz NG. Key facts about Asian Americans | Pew Research Center. https://www.pewresearch.org/fact-tank/2021/04/29/key-facts-about-asian-americans/. Published 2021. Accessed September 3, 2021.
2. Echeverria-Estrada C, Batalova J. Chinese Immigrants in the United States | migrationpolicy.org. https://www.migrationpolicy.org/article/chinese-immigrants-united-states-2018. Published 2020. Accessed September 3, 2021.
3. Rajpathak SN, Wylie-Rosett J. High prevalence of diabetes and impaired fasting glucose among chinese immigrants in New York City. *J Immigr Minor Heal*. 2011;13(1):181-183. doi:10.1007/s10903-010-9356-2
4. Thorpe LE, Upadhyay UD, Chamany S, et al. Prevalence and control of diabetes and impaired fasting glucose in New York City. *Diabetes Care*. 2009;32(1):57-62. doi:10.2337/dc08-0727
5. Islam NS, Wyatt LC, Kapadia SB, Rey MJ, Trinh-Shevrin C, Kwon SC. Diabetes and associated risk factors among Asian American subgroups in New York City. *Diabetes Care*. 2013;36(1):2013. doi:10.2337/dc12-1252
6. Islam NS, Kwon SC, Wyatt LC, et al. Disparities in diabetes management in Asian Americans in New York City compared with other racial/ethnic minority groups. *Am J Public Health*. 2015;105:S443-S446. doi:10.2105/AJPH.2014.302523
7. Ma RCW, Chan JCN. Type 2 diabetes in East Asians: similarities and differences with populations in Europe and the United States. *Ann N Y Acad Sci*. 2013;1281(1):64-91. doi:10.1111/nyas.12098
8. Lanting LC, Joung IMA, Mackenbach JP, Lamberts SWJ, Bootsma AH. Ethnic differences in mortality, end-stage complications, and quality of care among diabetic patients: a review. *Diabetes Care*. 2005;28(9):2280-2288. http://www.ncbi.nlm.nih.gov/pubmed/16123507. Accessed May 30, 2017.

9. Fan W, Lee DH, Billimek J, Choi S, Wang PH. The changing landscape of diabetes prevalence among first-generation Asian immigrants in California from 2003 to 2013. *BMJ Open Diabetes Res Care*. 2017;5(1):e000327. doi:10.1136/BMJDRC-2016-000327

10. King GL. Can Disparity of Care for Diabetes be overcome in Asian-Americans today? AANHPI diabetes disparities partnership forum. http://main.diabetes.org/dorg/disparities-partnership-fourm/day-2-aanhpi-session.pdf.

11. King GL, Mcneely MJ, Thorpe LE, et al. Understanding and addressing unique needs of diabetes in Asian Americans, Native Hawaiians, and Pacific Islanders. *Diabetes Care*. 2012;35(5):1181-1188. doi:10.2337/dc12-0210

12. Powers MA, Bardsley JK, Cypress M, et al. Diabetes Self-management Education and Support in Adults With Type 2 Diabetes: A Consensus Report of the American Diabetes Association, the Association of Diabetes Care & Education Specialists, the Academy of Nutrition and Dietetics, the American Academy of Family Physicians, the American Academy of PAs, the American Association of Nurse Practitioners, and the American Pharmacists Association. *Diabetes Care*. 2020;43(7):1636-1649. doi:10.2337/DCI20-0023

13. Chesla CA, Chun KM. Accommodating type 2 diabetes in the Chinese American family. *Qual Health Res*. 2005;15(2):240-255. doi:10.1177/1049732304272050

14. Chesla CA, Chun KM, Kwan CML. Cultural and family challenges to managing type 2 diabetes in immigrant Chinese Americans. *Diabetes Care*. 2009;32(10):1812-1816. doi:10.2337/dc09-0278

15. Chun KM, Chesla CA. Cultural issues in disease management for chinese americans with type 2 diabetes. *Psychol Health*. 2004;19(6):767-785. doi:10.1080/08870440410001722958

16. Maine Department of Health and Human Services. *Diabetes Self-Management Education Barrier Study*.; 2006.

17. Yeh M, Heo M, Suchday S, et al. Translation of the Diabetes Prevention Program for diabetes risk reduction in Chinese immigrants in New York City. *Diabet Med*. 2015;33:547-551. doi:10.1111/dme.12848

18. Wang Y, Chuang L, Bateman WB. Focus group study assessing self-management skills of Chinese Americans with Type 2 diabetes mellitus. *J Immigr Minor Heal*. 2012;14(5):869-874. doi:10.1007/s10903-011-9514-1

19. Mendoza-Herrera K. An Overview of Social Media Use in the Field of Public Health Nutrition: Benefits, Scope, Limitations, and a Latin American Experience. *Prev Chronic Dis*. 2020;17. doi:10.5888/PCD17.200047

20. Hong YA, Juon H-S, Chou W-YS. Social media apps used by immigrants in the United States: challenges and opportunities for public health research and practice. *mHealth*. 2020:0-0. doi:10.21037/MHEALTH-20-133

21. Williams G, Hamm MP, Shulhan J, Vandermeer B, Hartling L. Social media interventions for diet and exercise behaviours: A systematic review and meta-analysis of randomised controlled trials. *BMJ Open*. 2014;4(2). doi:10.1136/BMJOPEN-2013-003926

22. Gabarron E, Arsand E, Wynn R. Social media use in interventions for diabetes: Rapid evidence-based review. *J Med Internet Res*. 2018;20(8). doi:10.2196/10303

23. Korda H, Itani Z. Harnessing Social Media for Health Promotion and Behavior Change.

*Health Promot Pract*. 2013;14(1):15-23. doi:10.1177/1524839911405850

24. Mayberry LS, Lyles CR, Oldenburg B, Osborn CY, Parks M, Peek ME. mHealth Interventions for Disadvantaged and Vulnerable People with Type 2 Diabetes. *Curr Diab Rep*. 2019;19(12):148. doi:10.1007/s11892-019-1280-9

25. National Institute on Minority Health and Health Disparities. NIMHD Research Framework. https://www.nimhd.nih.gov/about/overview/research-framework/nimhd-framework.html. Published 2017. Accessed September 20, 2021.

26. Islam N, Zanowiak MJ, Riley L, Nadkarni KS, Kwon CS, Shervrin C. characteristics of Asian Amarican, native Hawaaiian, and Pacific Islander community health worker programs: a systematic review. *Heal care Poor Underserved*. 2015;26(2 0):1-22. doi:10.1353/hpu.2015.0062.Characteristics

27. Hill-Briggs F, Adler NE, Berkowitz SA, et al. Social Determinants of Health and Diabetes: A Scientific Review. *Diabetes Care*. 2021;44(1):258-279. doi:10.2337/DCI20-0053

28. Katigbak C, Au H, Zuo S, Chan P. Chinese immigrants' views on exercise and using technology to enhance physical activity. In: *International Association of Gerotology & Geriatrics*. San Francisco, California, USA; 2017. doi:10.1016/0749-5978(91)90020-T

29. Hu L, Trinh-Shevrin C, Yi SS, et al. Diabetes Management and Technology Use among Chinese Americans in NYC: Current Status and Future Opportunities. In: Poster Presentation at the 9th Biennial Asian American, Native Hawaiian, and Pacific Islander Health Conference, New York, NY; 2018.

30. Jiang N, Zhang Y, Qian X, Thorpe L, Trinh-Shevrin C, Shelley D. Chinese immigrant smokers' access barriers to tobacco cessation services and experience using social media and text messaging. *Tob Prev Cessat*. 2020;6(September):1-10. doi:10.18332/TPC/125942

31. Hu L, Islam N, Kharmats AY, et al. A Social Media-Based Intervention Improves Glycemic Control in a Low-Income Older Immigrant Population. *Diabetes*. 2021;70(Supplement 1):1-OR. doi:10.2337/DB21-1-OR

32. Davidson KW, Krist AH, Tseng C-W, et al. Incorporation of Social Risk in US Preventive Services Task Force Recommendations and Identification of Key Challenges for Primary Care. *JAMA*. September 2021. doi:10.1001/JAMA.2021.12833

33. Islam NS, Wyatt LC, Taher MD, et al. A Culturally Tailored Community Health Worker Intervention Leads to Improvement in Patient-Centered Outcomes for Immigrant Patients With Type 2 Diabetes. *Clin Diabetes*. 2018;36(2):100. doi:10.2337/CD17-0068

34. Lim S, Wyatt L, Chauhan H, et al. A Culturally Adapted Diabetes Prevention Intervention in the New York City Sikh Asian Indian Community Leads to Improvements in Health Behaviors and Outcomes. *Heal Behav Res*. 2019;2(1). doi:10.4148/2572-1836.1027

35. Chesla CA, Chun KM, Kwan CML, et al. Testing the efficacy of culturally adapted coping skills training for Chinese American immigrants with type 2 diabetes using community-based participatory research. *Res Nurs Heal*. 2013;36(4):359-372. doi:10.1002/nur.21543

36. Xu Y, Toobert D, Savage C, Pan W, Whitmer K. Factors influencing diabetes self-management in Chinese people with type 2 diabetes. *Res Nurs Health*. 2008;31(6):613-625. doi:10.1002/nur.20293

37. Lorig K, Ritter PL, Villa FJ, Armas J. Community-based peer-led diabetes self-management: A randomized trial. *Diabetes Educ*. 2009;35(4):641-651.

doi:10.1177/0145721709335006

38. Pauley T, Gargaro J, Chenard G, Cavanagh H, McKay SM. Home-based diabetes self-management coaching delivered by paraprofessionals: A randomized controlled trial. *http://dx.doi.org/101080/0162142420161264339*. 2017;35(3-4):137-154. doi:10.1080/01621424.2016.1264339

39. Beckerle CM, Lavin MA. Association of Self-Efficacy and Self-Care With Glycemic Control in Diabetes. *Diabetes Spectr*. 2013;26(3):172-178. doi:10.2337/DIASPECT.26.3.172

40. Lorig K, Ritter PL, Laurent DD, et al. Online Diabetes Self-Management Program. *Diabetes Care*. 2010;33(6):1275-1281. doi:10.2337/DC09-2153

41. Toobert D, Hampson S, Glasgow R. The summary of diabetes self-care activities measure: results from 7 studies and a revised scale. *Diabetes Care*. 2000;23(7):943-950. doi:10.2337/DIACARE.23.7.943

42. Yin Xu, Savage C, Toobert D, Wei Pan, Whitmer K. Adaptation and Testing of Instruments to Measure Diabetes Self-Management in People With Type 2 Diabetes in Mainland China. *J Transcult Nurs*. 2008;19(3):234-242. doi:10.1177/1043659608319239

43. Paxton AE, Strycker LA, Toobert DJ, Ammerman AS, Glasgow RE. Starting The Conversation. *Am J Prev Med*. 2011;40(1):67-71. doi:10.1016/j.amepre.2010.10.009

44. National Cancer Institute. Dietary Screener Questionnaire in the NHANES 2009-10: Background. https://epi.grants.cancer.gov/nhanes/dietscreen/. Accessed May 23, 2017.

45. Craig C, Marshall A, Sjöström M, et al. International Physical Activity Questionnaire: 12-Country Reliability and Validity. *Med Sci Sport Exerc*. 2003;35(8):1381-1395. doi:10.1249/01.MSS.0000078924.61453.FB

46. International physical activity questionnaire. 2002. http://uacc.arizona.edu/sites/default/files/ipaq_english_telephone_short.pdf. Accessed August 23, 2017.

47. Stephenson M. Development and Validation of the Stephenson Multigroup Acculturation Scale (SMAS). *Psychological*. 2000;12(I):77-88. doi:10.1037//1040-3590.12.1.77

48. Hamilton C, Strader L, Pratt J, et al. The PhenX Toolkit: Get the Most From Your Measures. *Am J Epidemiol*. 2011;174(3):253-260. https://www.ncbi.nlm.nih.gov/pmc/articles/PMC3141081/. Accessed July 8, 2020.

49. VanderWeele TJ. *Explanation in Causal Inference : Methods for Mediation and Interaction*. Oxford University Press; 1st edition; 2015.

50. VanderWeele TJ, Vansteelandt S. Mediation Analysis with Multiple Mediators. *Epidemiol Method*. 2014;2(1):95. doi:10.1515/EM-2012-0010

51. Vanderweele TJ, Vansteelandt S. Conceptual issues concerning mediation, interventions and composition. *Stat Interface*. 2009;2(4):457-468. doi:10.4310/SII.2009.V2.N4.A7

52. VanderWeele TJ. Bias formulas for sensitivity analysis for direct and indirect effects. *Epidemiology*. 2010;21(4):540-551. doi:10.1097/EDE.0B013E3181DF191C

53. Valeri L, Vanderweele TJ. SAS macro for causal mediation analysis with survival data. *Epidemiology*. 2015;26(2):e23-e24. doi:10.1097/EDE.0000000000000253

54. Valeri L, VanderWeele TJ. Mediation analysis allowing for exposure-mediator interactions and causal interpretation: theoretical assumptions and implementation with SAS and

SPSS macros. *Psychol Methods*. 2013;18(2):137-150. doi:10.1037/A0031034
