# Supplementary material for: Leveraging videos and community health workers to address social determinants of health in immigrants (LINK-IT): Protocol for a randomized controlled trial
Source: PLoS One. 2026 Feb 2;21(2):e0341217. doi: 10.1371/journal.pone.0341217 (PMC12863526; doi:10.1371/journal.pone.0341217)
Supplement: Attachment — Submitted filename: 0. Response Letter_PlosOne 22.12.2025.docx [file pone.0341217.s004.docx]

**Re: Response Letter**

PONE-D-25-64951

**Date**: 22 Dec 2025

**Leveraging Videos and Community Health Workers to Address Social Determinants of Health in Immigrants (LINK-IT): Protocol for a Randomized Controlled Trial**

Dear Editors,

Thank you for giving us the opportunity to revise our manuscript. We have carefully reviewed your comments and uploaded all the requested materials, including:

- The NIH funding letter;
- IRB extension covering data recruitment and collection;
- The study protocols submitted to the IRB and the approved study protocol with detailed project information and data analysis plans.

Additionally, we have revised the manuscript to include information on the verbal informed consent and confirmed that it has been approved by the IRB.

We sincerely appreciate your consideration and look forward to feedback from the editors and reviewers.

Sincerely,

Lu Hu, PhD

Associate Professor
Department of Population Health
NYU Grossman School of Medicine, NYU Langone Health

180 Madison Ave, New York, NY 10016
